# Supplementary figures and images for: Diversity and efficacy of insect sampling methods in an urban tropical dry forest of the Colombian Caribbean
Source: PeerJ. 2025 Feb 26;13:e18262. doi: 10.7717/peerj.18262 (PMC11871896; doi:10.7717/peerj.18262)

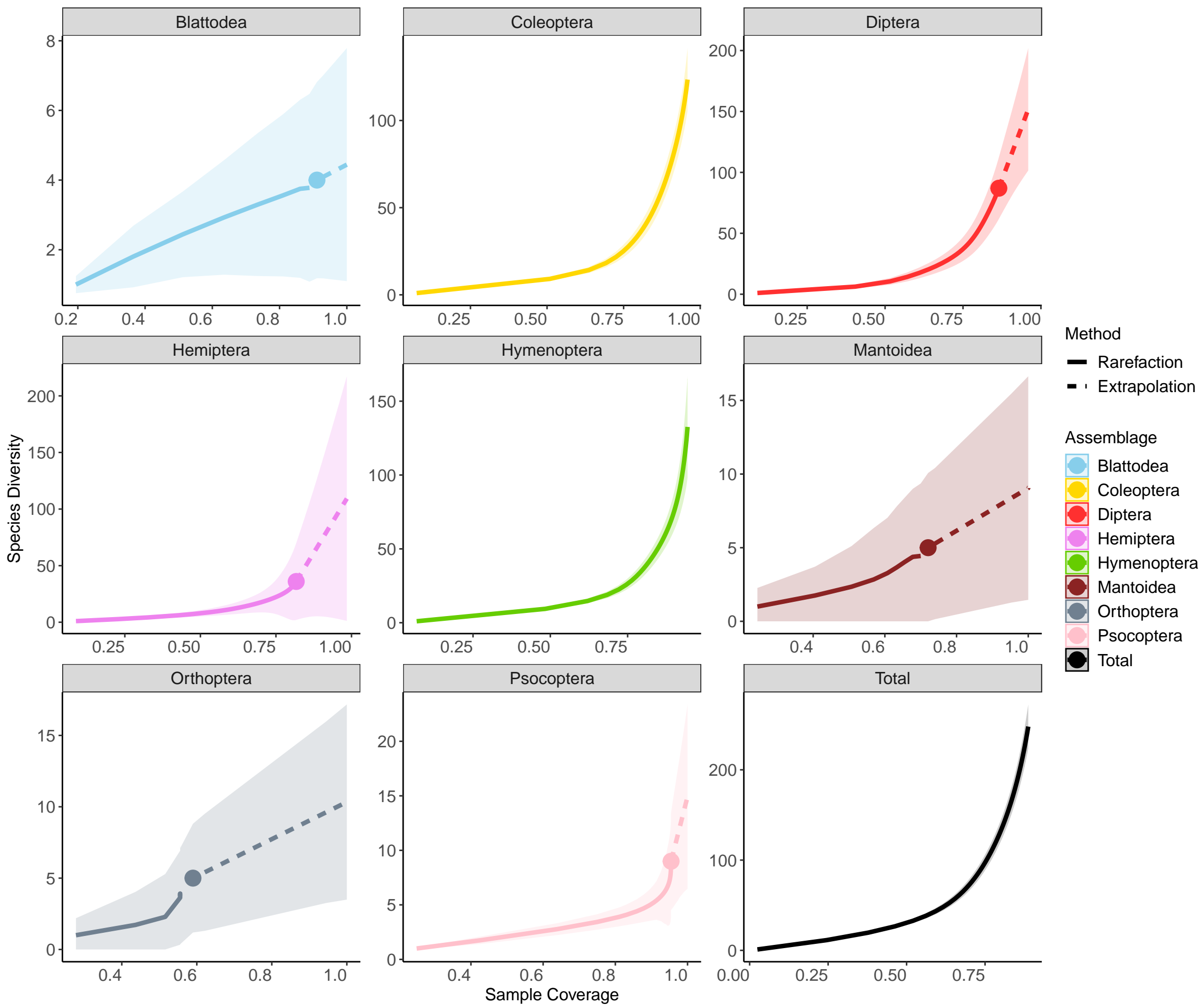

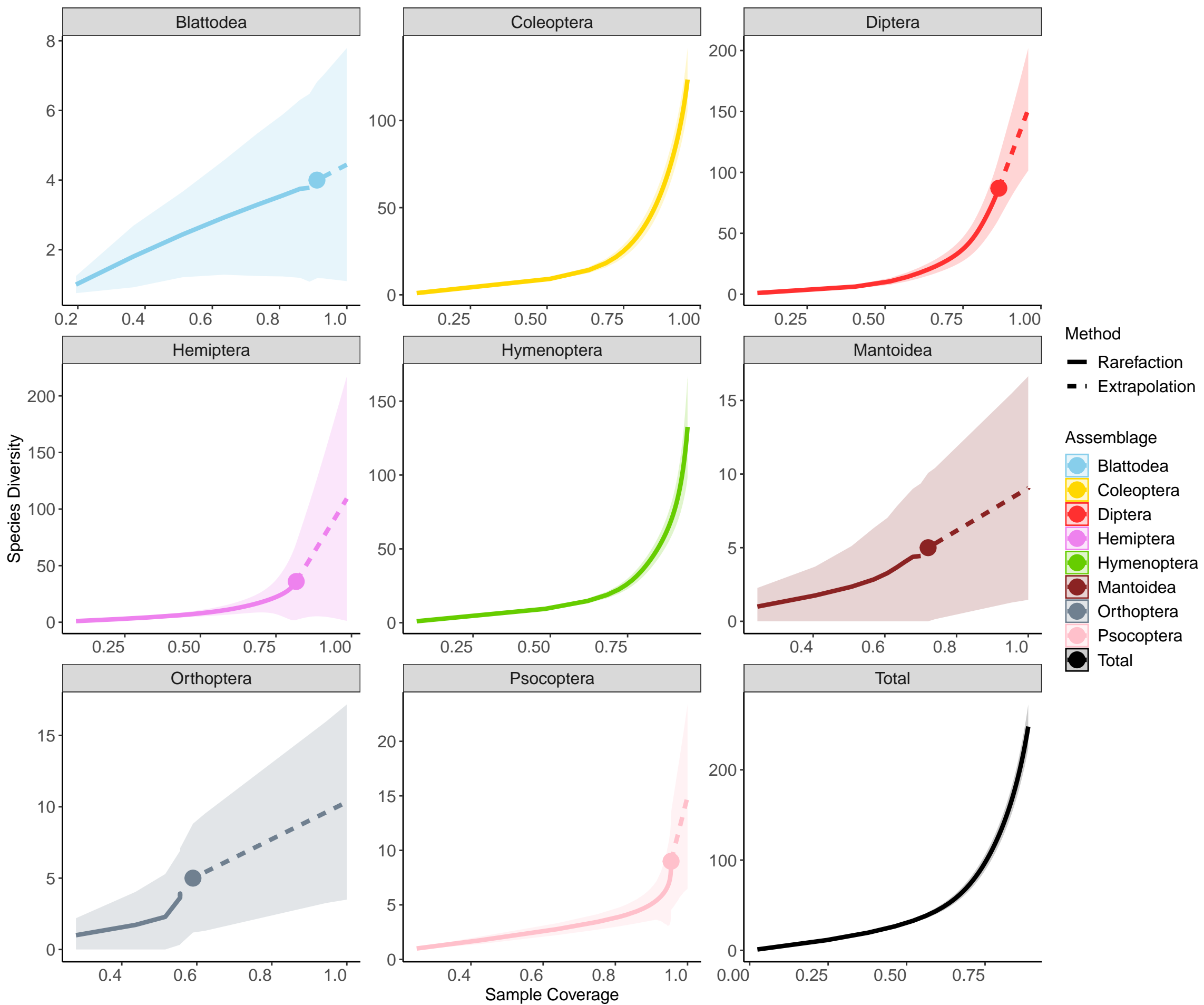

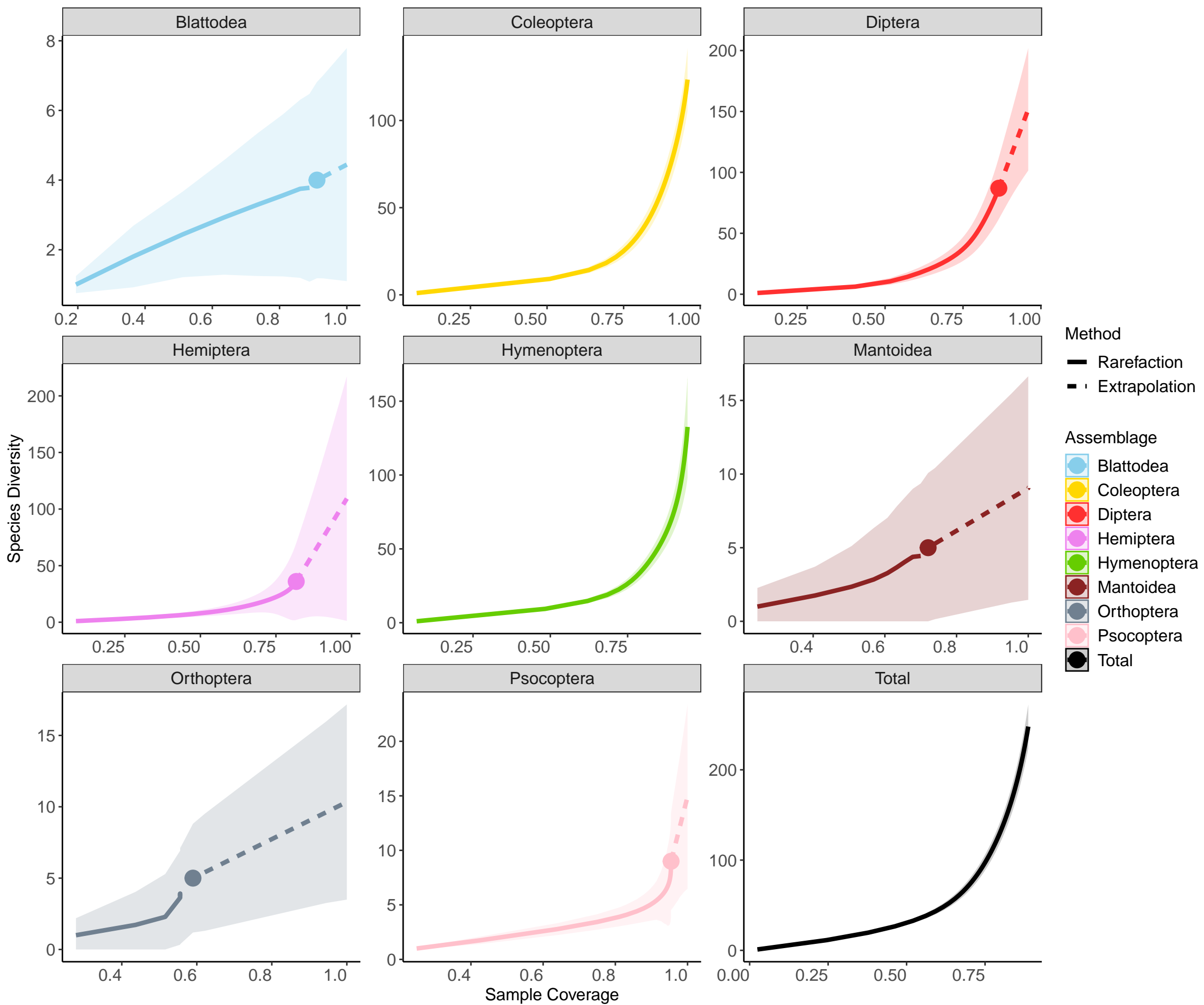

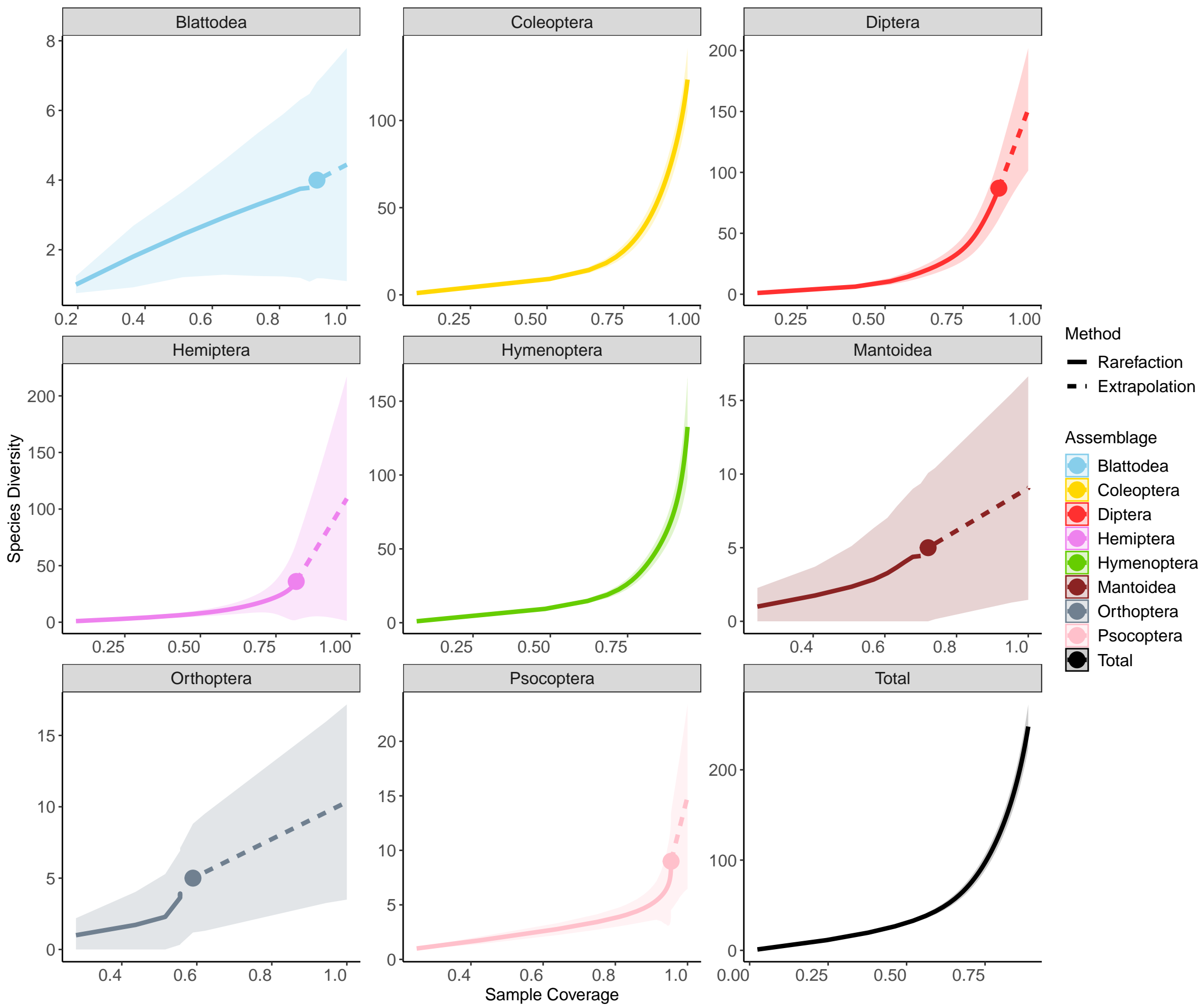

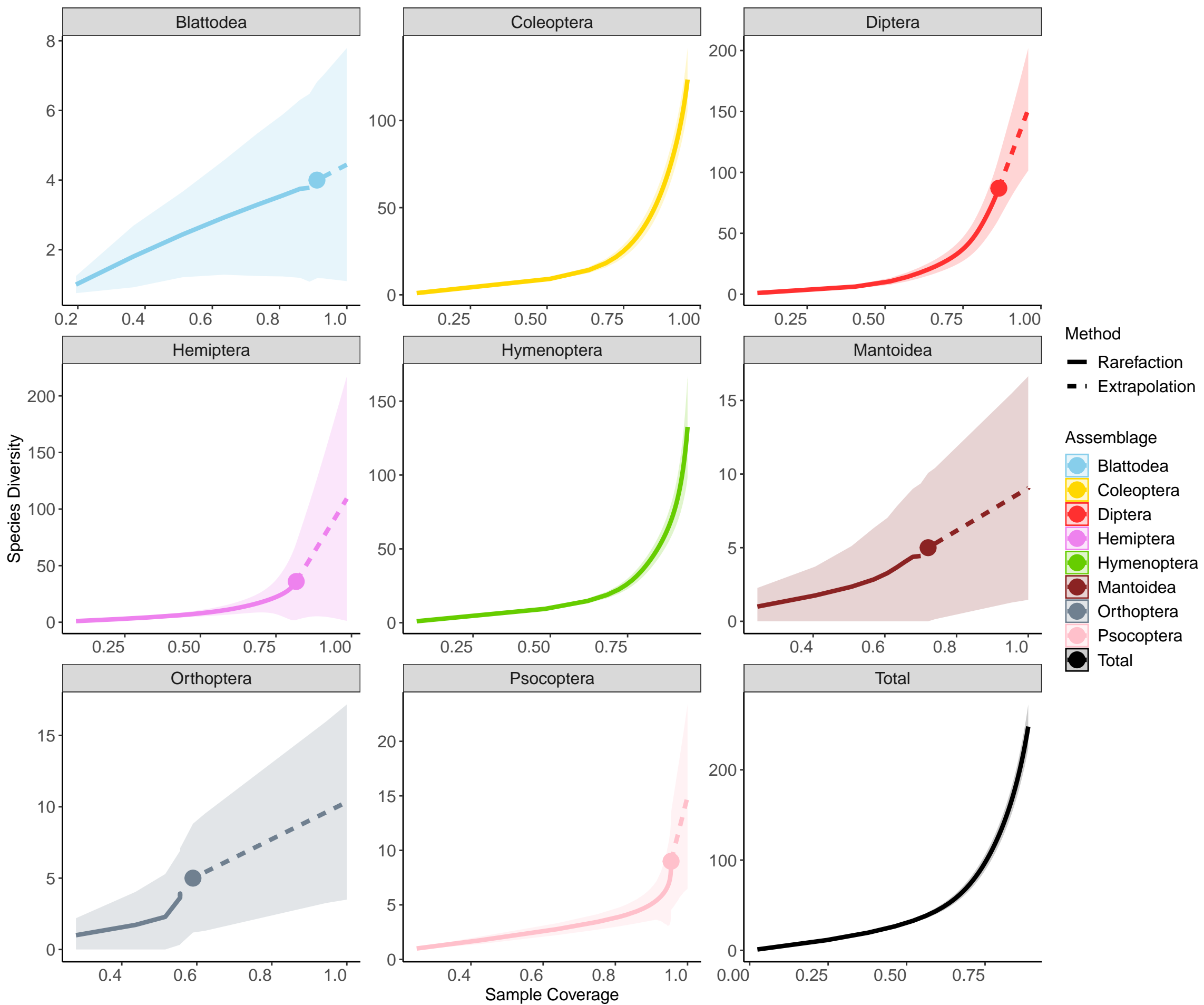

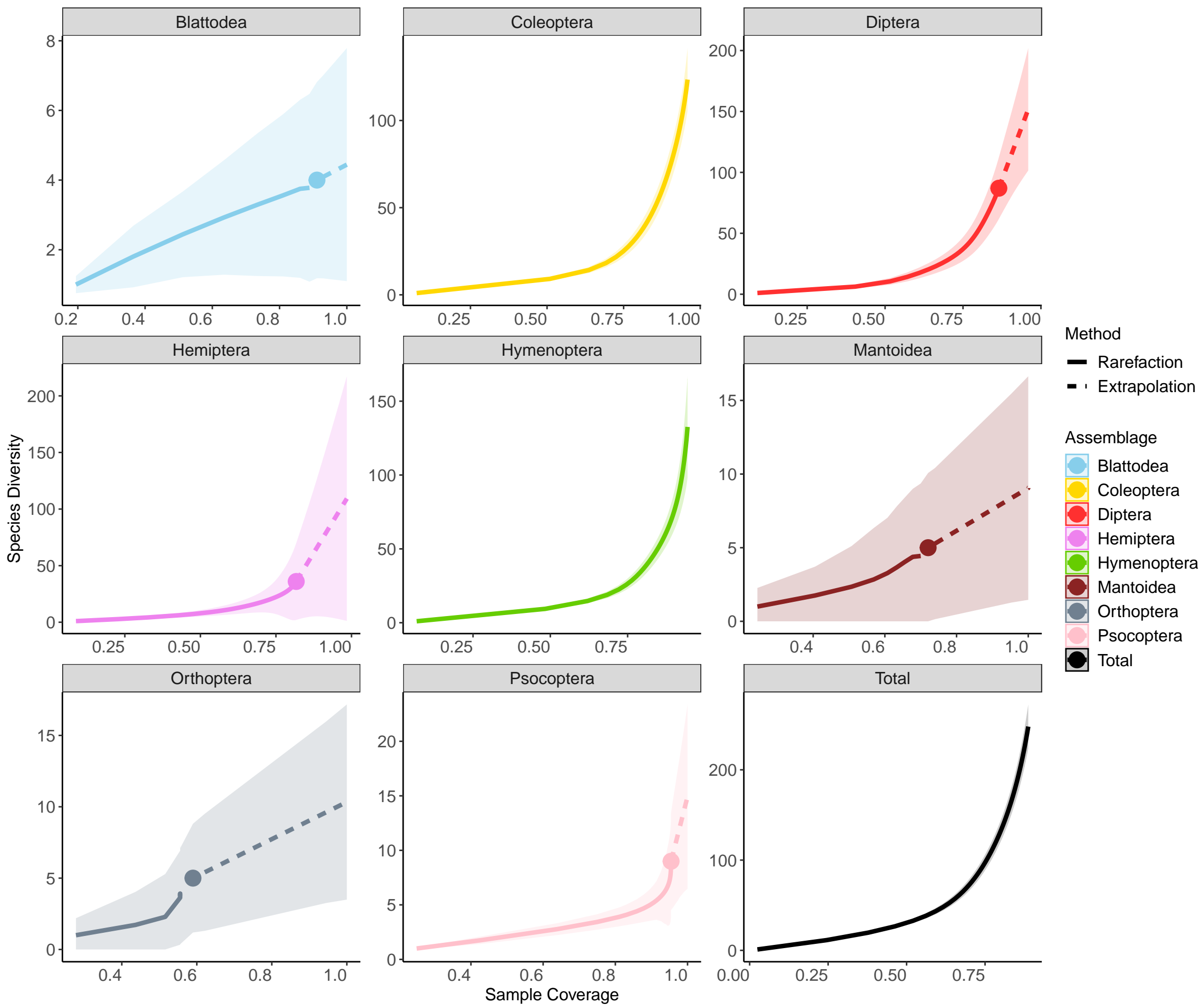

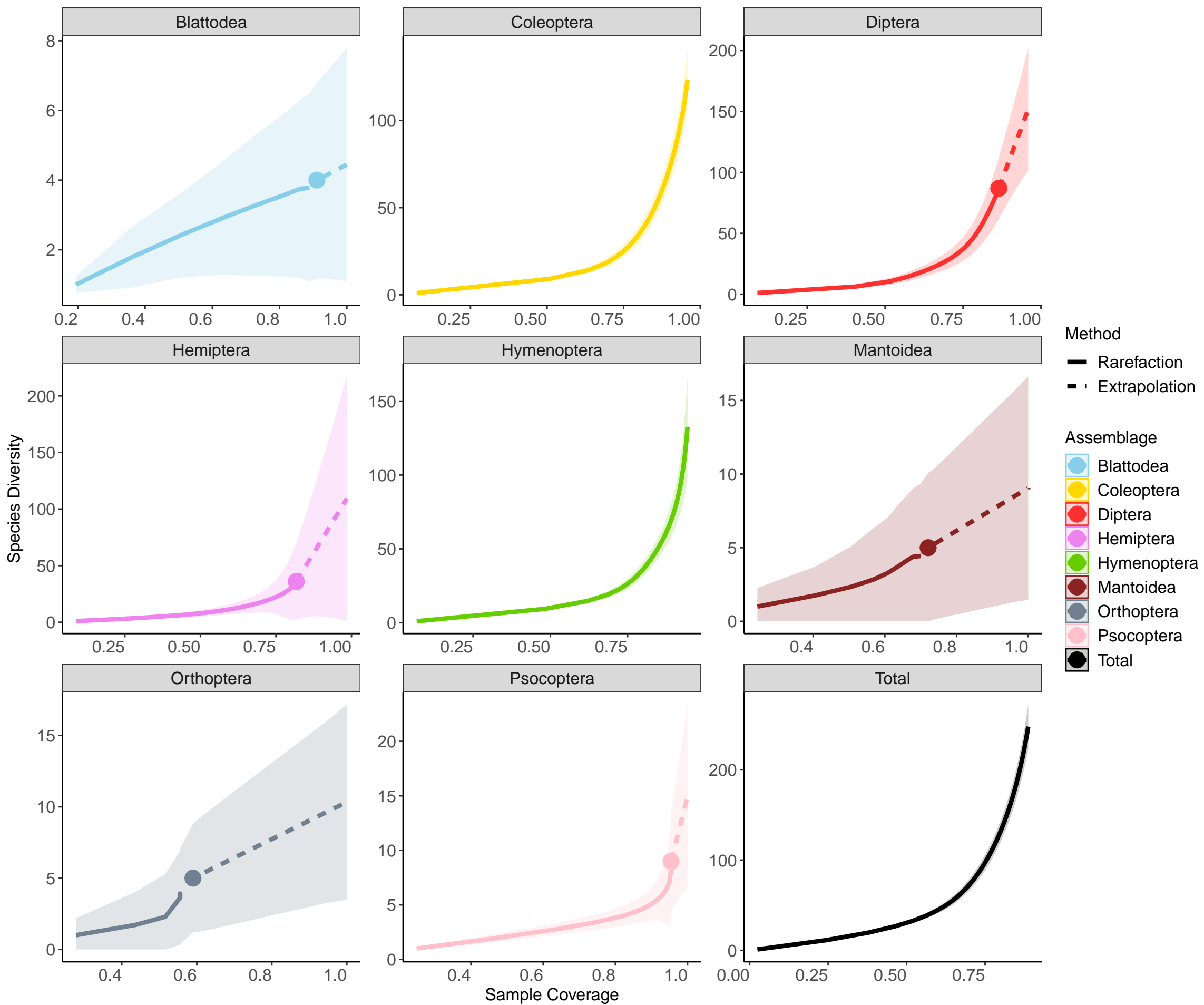

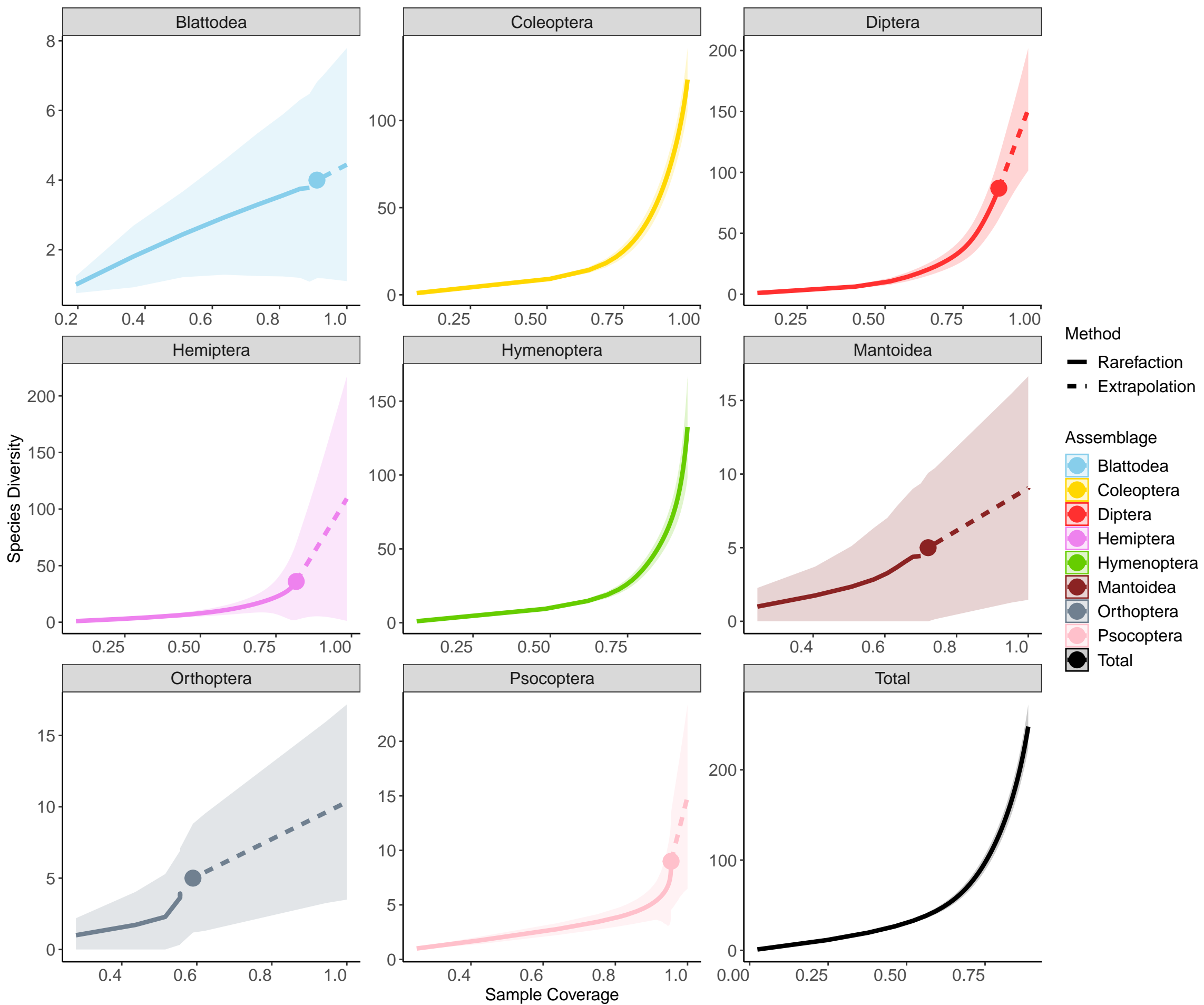

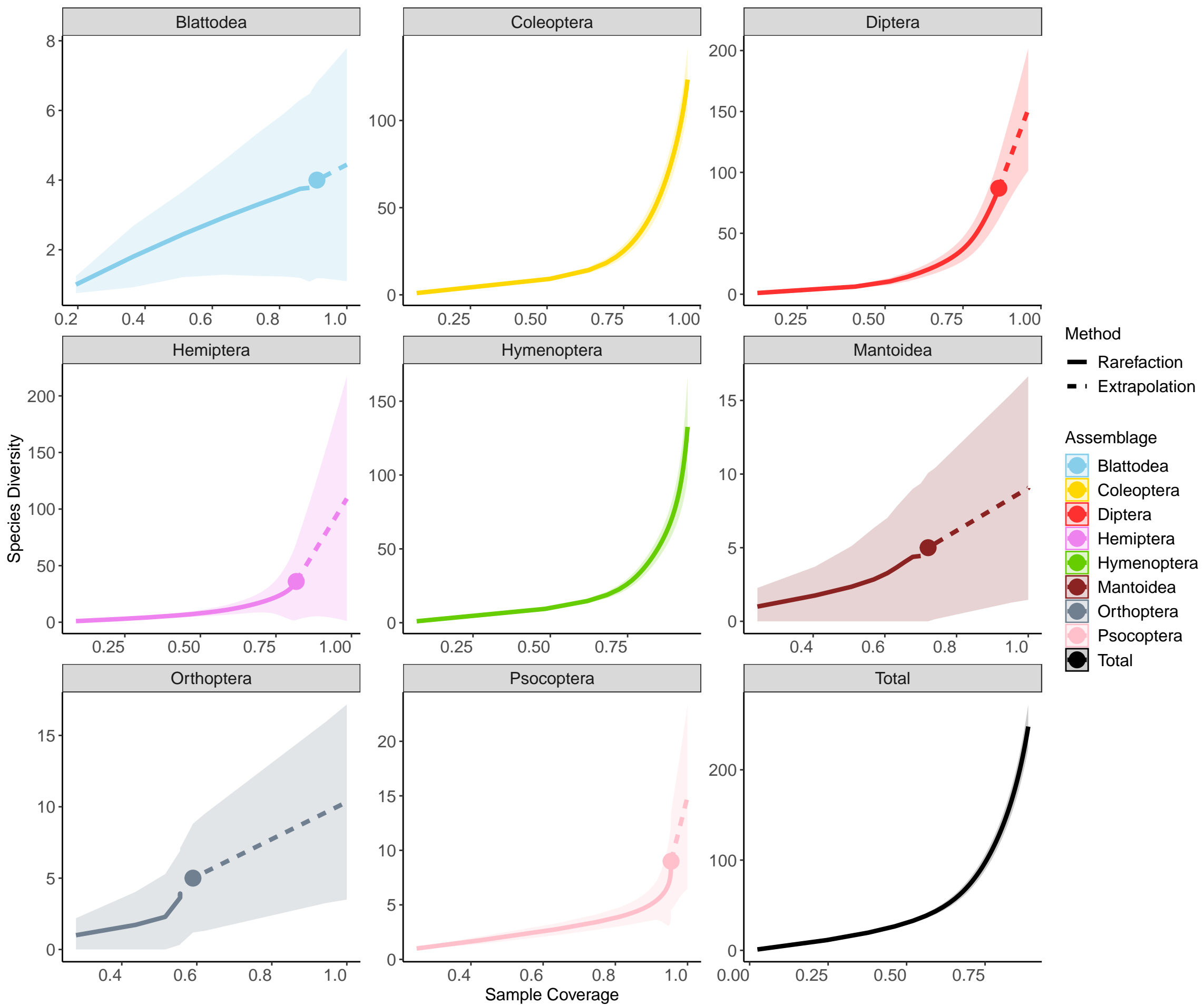

Supplement: Supplemental Information 3 — Each line represents the dynamic behavior of the richness relative to the total sampling. Continuous line represents rarefaction, while dotted line represents extrapolation. Symbol indicates estimated richness. Only most divers orders shown, with Coleoptera, Diptera and Hymenoptera having well behaved curves due to larger sample sizes. All other orders have much smaller sample sizes. [file peerj-13-18262-s003.pdf]
